# Risks Involved in the Use of Enrofloxacin for *Salmonella* Enteritidis or *Salmonella* Heidelberg in Commercial Poultry

**DOI:** 10.3389/fvets.2016.00072

**Published:** 2016-08-31

**Authors:** Eduardo Morales-Barrera, Nicole Calhoun, Jose L. Lobato-Tapia, Vivian Lucca, Omar Prado-Rebolledo, Xochitl Hernandez-Velasco, Ruben Merino-Guzman, Victor M. Petrone-García, Juan D. Latorre, Brittany D. Mahaffey, Kyle D. Teague, Lucas E. Graham, Amanda D. Wolfenden, Mikayla F. A. Baxter, Billy M. Hargis, Guillermo Tellez

**Affiliations:** ^1^Departamento de Producción Agrícola y Animal, Universidad Autónoma Metropolitana, Mexico City, México; ^2^Department of Poultry Science, University of Arkansas, Fayetteville, AR, USA; ^3^Departamento de Medicina Veterinaria, Centro de Ciencias Rurais, Universidade Federal de Santa Maria, Santa Maria, Brazil; ^4^Facultad de Medicina Veterinaria y Zootecnia, Universidad de Colima, Colima, México; ^5^Facultad de Medicina Veterinaria y Zootecnia, Universidad Nacional Autónoma de México, Ciudad de México, México; ^6^Departamento de ciencias pecuarias, Facultad de Estudios Superiores Cuautitlán UNAM, Cuautitlán, México

**Keywords:** Enrofloxacin, *Salmonella*, poultry, susceptibility, metagenomics

## Abstract

The objectives of the present study were to evaluate the risks involved in the use of Enrofloxacin for *Salmonella* Enteritidis (SE) or *Salmonella* Heidelberg (SH) in commercial poultry and determine the effects of a probiotic as an antibiotic alternative. Two experiments were conducted to evaluate the risks involved in the use of Enrofloxacin for SE or SH in commercial poultry. Experiment 1 consisted of two trials. In each trial, chickens were assigned to one of three groups; control + SE challenged; Enrofloxacin 25 mg/kg + SE; and Enrofloxacin 50 mg/kg + SE. Chickens received Enrofloxacin in the drinking water from days 1 to 5 of age. On day 6, all groups received fresh water without any treatment. All chickens were orally gavaged with 10^7^ cfu/chick of SE at 7 days of age and euthanized on 8 days of age. In Experiment 2, turkey poults were assigned to one of the three groups; control + SH; probiotic + SH; and Enrofloxacin 50 mg/kg + SH. Poults received probiotic or Enrofloxacin in the drinking water from days 1 to 5 of age. On day 6, poults received fresh water without any treatment. Poults were orally gavaged with 10^7^ cfu/poult of SH at 7 days of age. Poults were weighed and humanely killed 24 h post-SH challenge to evaluate serum concentration of fluorescein isothiocyanate-dextran to evaluate intestinal permeability, metagenomics, and SH infection. In both trials of Experiment 1, chickens treated with Enrofloxacin were more susceptible to SE organ invasion and intestinal colonization when compared with control non-treated chickens (*P* < 0.05). In Experiment 2, poults treated with 50 mg/kg of Enrofloxacin showed an increase in body weight, however, this group also showed an increase in SH susceptibility, intestinal permeability, and lower proportion of Firmicutes and Bacteroidetes, but with control group had the highest proportion of Proteobacteria. By contrast, poults that received the probiotic had the highest proportion of Firmicutes and Bacteroidetes, but lowest Proteobacteria. The results of the present study suggest that prophylactic utilization of Enrofloxacin at five times the recommended dose in poultry increases the susceptibility to salmonellae infections, and confirms that probiotics may be an effective tool in salmonellae infections.

## Introduction

Fluoroquinolones are the third generation of quinolone development. Nalidixic acid and pipemidic acid are examples of the first generation and currently have limited activity against Gram-negative bacteria. Fluorinated 4-quinolones were introduced to the market in the 1980s and were the top of the line antibiotics, offering a broad spectrum of activity and high efficacy in a wide range of infections both orally and parenterally (1, 2). Nevertheless, history has demonstrated that the extensive use of new antibiotics is eventually shadowed by the appearance of resistance to those chemicals that have become a major global problem. This was demonstrated by the higher incidence of salmonellae and *Campylobacter* infections worldwide, and several reports of fluoroquinolone resistance in clinical isolates for these and other enteric pathogens (3–7). Hence, the World Health Organization (WHO) published a list of antibiotics that should be reserved for human use only (8), and fluoroquinolones were among them, due to the alarming evidence of quinolone-resistant zoonotic pathogens. Soon after the publication of the WHO report, several countries banned the use of fluoroquinolones in animal production (9–11). With growing consumer and scientific pressures, the European Union went one step further, creating new legislations banning the use of all antibiotics as growth promoters as of January 2006 (12). However, in many countries, the indiscriminate use and misuse of antibiotics, including fluoroquinolones, are still a sad reality. Especially in countries where there is no legislation regulating the use of fluoroquinolones in animal agriculture and where there is an abundance of generic fluoroquinolones at a low cost. Typical management practices in those countries are to treat or dose healthy neonatal chickens and turkey poults with five times the recommended dose of Enrofloxacin for five consecutive days in the drinking water. Interestingly, in those countries, the incidence of *Salmonella* spp. and *Campylobacter* spp. rates in both humans and agriculture are also high (1, 13–16). Therefore, the objectives of the present study were to evaluate and confirm the risks involved in the use of Enrofloxacin for *Salmonella* enterica serovars Enteritidis or Heidelberg in commercial poultry and to determine if poultry selected probiotics have a prophylactic effect when birds are challenged with SE and SH.

## Materials and Methods

### Enrofloxacin

Baytril^®^ (Bayer Health Care LLC, Mission, KS 66201, USA) Enrofloxacin 3.23% concentrate solution for use in chickens and turkeys drinking water only.

### Probiotic Culture

FloraMax^®^-B11 (Pacific Vet Group USA Inc., Fayetteville, AR 72703, USA) is a defined probiotic culture derived from gastrointestinal poultry origin that contains proprietary strains of lactic acid bacteria (LAB), selected by their *in vitro* ability to inhibit enteropathogens (17).

### Animal Source

Day-of-hatch, male broiler chickens were obtained from Cobb-Vantress (Siloam Springs, AR, USA) for Experiment 1 or male turkey poults from a local hatchery in Experiment 2 and were randomly housed in heated brooder batteries in a controlled age-appropriate environment. For each experiment, birds were provided *ad libitum* access to water, and unmedicated corn–soybean diet, meeting the nutritional requirements of poultry recommended by National Research Council (18), respectively. All animal handling procedures were in compliance with Institutional Animal Care and Use Committee at the University of Arkansas. In each experiment, a small number of chicks or poults (*n* = 10) were humanely euthanized upon arrival by CO_2_ asphyxiation. Ceca-cecal tonsils (CCT), liver, and spleen were aseptically cultured in tetrathionate enrichment broth (Catalog no. 210420, Becton Dickinson, Sparks, MD, USA). Enriched samples were confirmed negative for *Salmonella* by streak plating the samples on Xylose Lysine Tergitol-4 (XLT-4, Catalog no. 223410, BD Difco™) selective media.

### Bacterial Strains and Culture Conditions

The challenge organism used in Experiment 1 was a poultry isolate of *Salmonella enterica* serovar Enteritidis (SE), bacteriophage type 13A, obtained from the USDA National Veterinary Services Laboratory, Ames, IA, USA. In Experiment 2, a primary poultry isolate of *Salmonella enterica* serovar Heidelberg (SH) isolated in our laboratory was used. Antimicrobial susceptibility test revealed that both isolates were sensitive to Enrofloxacin. Furthermore, SE and SH are resistant to 25 μg/mL of novobiocin (NO, catalog no.N-1628, Sigma) and were selected for resistance to 20 μg/mL of nalidixic acid (NA, catalog no.N-4382, Sigma) in our laboratory. For both experiments, 100 μL of SE or SH from a frozen aliquot was added to 10 mL of tryptic soy broth (Catalog no. 22092, Sigma) and incubated at 37°C for 8 h, and passed three times every 8 h to ensure that all bacteria were in log phase. Post-incubation, bacterial cells were washed three times with sterile 0.9% saline by centrifugation at 1,864 × *g* for 10 min, reconstituted in saline, quantified by densitometry with a spectrophotometer (Spectronic 20D+, Spectronic Instruments Thermo Scientific), and diluted to an approximate concentration of 10^8^ cfu/milliliter. Concentrations of SE or SH were further verified by serial dilution and plating on brilliant green agar (BGA, Catalog no. 70134, Sigma) with NO and NA for enumeration of actual cfu used to challenge the chickens and turkeys.

### Experimental Design in Chickens and Turkeys

#### Evaluation of Enrofloxacin in Neonatal Chickens Challenged with *Salmonella* Enteritidis. Experiment 1

Two independent trials were conducted. In each trial, 36 chickens were randomly assigned to one of three groups (*n* = 12): control SE challenged without Enrofloxacin; Enrofloxacin 25 mg/kg SE challenged; and Enrofloxacin 50 mg/kg SE challenged. Chickens received Enrofloxacin from days 1 to 5 of age in the drinking water. At day 6, treated groups received fresh water without any treatment. Fresh water without antibiotic was administered to control chickens throughout the experiment. All chickens were orally gavaged with 10^7^ cfu/chick of SE at 7 days of age. Chickens were humanely euthanized for culture at 8 days of age as describe below.

##### *Salmonella* Recovery

At 8 days, broilers were humanely euthanized and liver and spleen were collected aseptically and enriched in 10 mL of tetrathionate broth (Becton Dickinson) overnight at 37°C. Following enrichment, each sample was streaked for isolation on BGA plates containing 25 μg/mL of NO and 20 μg/mL of NA. The plates were incubated at 37°C for 24 h and examined for the presence or absence of antibiotic-resistant SE. CCT were collected aseptically, homogenized within sterile sample bags (Nasco, Fort Atkinson, WI, USA) using a rubber mallet and diluted with saline (1:4 by wt/vol) and 10-fold dilutions were plated on BGA with NO and NA, incubated at 37°C for 24 h to enumerate total SE colony forming units. The CCT samples were enriched in 2× concentrated tetrathionate enrichment broth and further incubated at 37°C for 24 h to enrich. Following this, enrichment samples were plated on BGA with NO and NA and incubated at 37°C for 24 h to confirm presence/absence of typical lactose-negative colonies of *Salmonella*.

#### Evaluation of Prophylactic Administration of FloraMax-B11^®^ Enrofloxacin in Neonatal Turkey Poults Challenged with *Salmonella* Heidelberg. Experiment 2

In Experiment 2, 72 day-of-hatch turkey poults were neck tagged, weighed, and randomly assigned to one of the three groups (*n* = 24/group): control SH challenged without treatment; probiotic SH challenged; and Enrofloxacin 50 mg/kg SH challenged. Poults received FloraMax-B11^®^ or Enrofloxacin from days 1 to 5 of age in the drinking water. Control group received fresh water without any treatment throughout the duration of the experiment. At day 6, treated groups received water without any treatment. All poults were orally gavaged with 10^7^ cfu/poult of SH at 7 days of age. Poults were weighed and humanely euthanized 24 h post-SH challenge (day 8 of age) to evaluate serum concentration of fluorescein isothiocyanate-dextran (FITC-D) and cecal bacterial community compositions as describe below, as well as *Salmonella* recovery and plating from CCT as was previously described. Samples from CCT were also plated in Man Rogosa Sharpe (Difco™ Lactobacilli MRS Agar VWR Cat. No. 90004-084 Suwanee, GA 30024) to evaluate total number of LAB.

##### Serum Determination of FITC-D Leakage

Intestinal leakage of FITC-D (MW 3–5 KDa; Sigma-Aldrich Co., St. Louis, MO, USA) and the measurement of its serum concentration were done in experiment 2 as a marker of paracellular transport and mucosal barrier dysfunction (19–22). At 24 h, post-SH challenge (day 8 of age), poults in all groups were given an oral gavage dose of FITC-D (4.16 mg/kg). Following 2.5 h, they were killed by CO_2_ asphyxiation. Blood samples were collected from the femoral vein kept at room temperature for 3 h and centrifuged (500 × *g* for 15 min) to separate the serum from the red blood cells. FITC-D levels of diluted serum samples (1:5 PBS) were measured at excitation wavelength of 485 nm and an emission wavelength of 528 nm with a Synergy HT, Multi-mode microplate fluorescence reader (BioTek Instruments, Inc., Vermont, USA). Fluorescence measured was then compared to a standard curve with known FITC-D concentrations. Gut leakage for each bird was reported as microgram of FITC-D/mL of serum (20).

##### DNA Extraction and Illumina-Based Analysis of Microbial Community Diversity

Cecal content from six poults was obtained, homogenized thoroughly in four volumes diluent (0.85% NaCl, 0.1% peptone), centrifuged at 300 × *g* for 2 min to remove large debris, and finally, 0.5 mL of aliquots (average 8 mg dry weight) were pelleted at 10,000 × *g* for 5 min. Extraction of DNA was performed immediately using the QIAamp DNA Stool Mini Kit (QIAGEN, Hilden, Germany). Bacterial community compositions at Phylum and Class level were performed using Illumina dye sequencing (Era7 Bioinformatics Inc., Cambridge, MA 02142, USA). The analysis corresponded to 16S rRNA amplicons from V6 region sequenced with Illumina technology (23). Reads were assigned to a taxon based on sequence similarity to 16S rRNA genes extracted from the NCBI nt database. The 16S rRNA sequences were extracted from NCBI based on their presence in the set of sequences included in the Ribosomal Database Project (RDP) (24) and on the specificity of their taxonomical assignment based on the lowest common ancestor (LCA) approach adopted metagenomics analysis as the last version of Meta-Genome Analyzer (MEGAN). The algorithm was similar to the assignment algorithm adopted by MEGAN tool (25). Phylum distribution in all the samples is expressed in % on the total merged reads of each sample.

### Data and Statistical Analysis

Log_10_ cfu/g of SE and SH in cecal contents, body weight (BW), body weight gain (BWG), serum FITC-D concentration, and proportion of bacterial composition were subjected to analysis of variance as a completely randomized design, using the General Linear Models procedure of SAS (26). Significant differences among the means were determined by Duncan’s multiple-range test at *P* < 0.05. Enrichment data were expressed as positive/total chickens (%), and the percent recovery of SE and SH was compared using the chi-squared test of independence, testing all possible combinations to determine the significance (*P* ≤ 0.05) for these studies (27).

## Results

The results from experiment 1, evaluating the effect of Enrofloxacin on neonatal chickens challenged with SE 24 h after antibiotic treatment on organ invasion and cecal colonization, are summarized in Table 1. In trial 1, there was a significant (*P* < 0.05) increase in the incidence of SE in liver and spleen in chickens treated with either 25 or 50 mg/kg of Enrofloxacin when compared with control chickens. Furthermore, chickens treated with 50 mg/kg of Enrofloxacin showed a 3.23 log increased in the incidence of SE in CCT as well as total cfu of SE/gram of ceca content when compared with control chickens and 0.45 log increase when compared with chickens treated with 25 mg/kg of Enrofloxacin. Similar results were observed in trial 2, where chickens treated with both doses of Enrofloxacin showed an increase in SE incidence in CCT as well as total numbers of SE in the cecal content when compared with control non-treated chickens (Table 1).

**Table 1:** **Evaluation of Enrofloxacin in neonatal chickens challenged with *Salmonella* Enteritidis (SE)^a^ 24 h after antibiotic treatment on organ invasion and cecal colonization**.

	Liver and spleen^b^	Log _10_ SE g/CCT^c^	Cecal tonsils^b^
**Trial 1**			
Control + SE	0/12 (0%)^e^	1.23 ± 0.45^e^	5/12 (41.7%)^e^
Enrofloxacin 25 mg/kg + SE	4/12 (33.3%)^d^	2.01 ± 0.66^e^	6/12 (50%)^e^
Enrofloxacin 50 mg/kg + SE	5/12 (41.7%)^d^	4.46 ± 0.37^d^	12/12 (100%)^d^
**Trial 2**			
Control + SE	0/12 (0%)^e^	1.23 ± 0.45^e^	5/12 (41.7%)^e^
Enrofloxacin 25 mg/kg + SE	4/12 (33.3%)^d^	2.01 ± 0.66^e^	6/12 (50%)^e^
Enrofloxacin 50 mg/kg + SE	5/12 (41.7%)^d^	4.46 ± 0.37^d^	12/12 (100%)^d^

*Experiment 1*.

*^a^Chickens received Enrofloxacin from days 1 to 5 of age in the drinking water. At day 6, all groups received fresh water without any treatment. All chickens were orally gavaged with 10^7^ cfu/chick of SE at 7 days of age. Chickens were humanely killed for culture at 8 days of age*.

*^b^Data of liver and spleen or ceca-cecal tonsils is expressed as positive/total chickens (%)*.

*^c^Log _10_ SE/g of ceca-ceca tonsils (CCT) data is expressed as mean ± SD*.

*^d,e^Superscripts within columns indicate significant difference at P < 0.05*.

The results from experiment 2 evaluating the prophylactic administration of FloraMax-B11^®^ or Enrofloxacin on organ invasion and cecal colonization of SH in neonatal turkey poults are summarized in Table 2. No significant differences were observed in the SH organ invasion between treated or control groups (*P* > 0.05), nevertheless, poults treated with the probiotic showed a significant reduction in both incidence of SH in CCT and total numbers of SH in ceca content when compared with poults treated with 50 mg/kg of Enrofloxacin or control non-treated poults (*P* < 0.05). Enrofloxacin poults also had a significant reduction in the total numbers of LAB (Table 2).

**Table 2:** **Evaluation of prophylactic administration of FloraMax-B11^®^ or Enrofloxacin on organ invasion and cecal colonization of *Salmonella* Heidelberg (SH)^a^ in neonatal turkey poults**.

	Liver and spleen^b^	Cecal tonsils^b^	Log_10_ SH/g of CCT^c^	Log_10_ Lactic acid bacteria/g of CCT^c^
Control SH	2/24 (8.33%)^d^	5/24 (20.83%)^d^	0.66 ± 0.29^d^	6.67 ± 0.26^d^
FloraMax-B11^®^ + SH	0/24 (0%)^d^	0/24 (0%)^e^	0.0 ± 0.0^e^	7.16 ± 0.24^d^
Enrofloxacin 50 mg/kg + SH	0/24 (0%)^d^	8/24 (33.33%)^d^	1.95 ± 0.28^d^	4.06 ± 0.22^e^

*Experiment 2*.

*^a^Poults received FloraMax-B11^®^ or Enrofloxacin from days 1 to 5 of age in the drinking water. At day 6, all groups received fresh water without any treatment. All poults were orally gavaged with 10^7^ cfu/poult of SH at 7 days of age. Poults were humanely killed for culture at 8 days of age*.

*^b^Data of liver and spleen as well as cecal tonsils is expressed as positive/total poults (%)*.

*^c^Log _10/_g of ceca-cecal tonsil (CCT) data is expressed as mean ± SD, *n* = 12*.

*^d,e^Superscripts within columns indicate significant difference at P < 0.05*.

The results of the evaluation of prophylactic administration of FloraMax-B11^®^ or Enrofloxacin on BW, BWG, and serum concentration of FITC-D in neonatal turkey poults in Experiment 2 are summarized in Table 3. Poults treated with 50 mg/kg of Enrofloxacin showed a significant increase in BW and BWG when compared with probiotic or control non-treated poults. Interestingly, poults in this group also showed a significant increase in gut permeability (Table 3).

**Table 3:** **Evaluation of prophylactic administration of FloraMax-B11^®^ or Enrofloxacin on body weight, body weight gain, and serum concentration of FITC-D^a^ in neonatal turkey poults**.

	Body weight (grams)	Body weight gain (grams)	Serum FITC-D (μg/mL)
Control SH	105.59 ± 2.31^c^	51.14 ± 2.45^c^	1.24 ± 0.08^c^
FloraMax-B11^®^ + SH	106.54 ± 2.24^c^	52.15 ± 2.39^c^	0.23 ± 0.06^c^
Enrofloxacin 50 mg/kg + SH	120.57 ± 2.60^b^	63.87 ± 2.71^b^	7.28 ± 3.09^b^

*Experiment 2*.

*^a^Poults received FloraMax-B11^®^ or Enrofloxacin from days 1 to 5 of age in the drinking water. At day 6, all groups received fresh water without any treatment. All poults were orally gavaged with 10^7^ cfu/poult of SH at 7 days of age. FITC-D was administered on 8 days of age*.

*^b,c^Superscripts within columns indicate significant difference at P < 0.05, n = 24*.

Table 4 shows the results of the Phylum distribution (cumulative% LCA) and class direct assignment in % for all ceca samples of turkey poults following prophylactic administration of FloraMax-B11^®^ or Enrofloxacin in Experiment 2. At the phylum level microbiome analysis, poults treated with the probiotic had the higher proportion of Firmicutes, followed by control poults and poults treated with Enrofloxacin. A significant reduction was observed in Bacteroidetes in poults treated with the antibiotic. Furthermore, significant increases in the proportion of Proteobacteria were observed in poults that received Enrofloxacin or control poults when compared with poults that received FloraMax-B11^®^. At the class level, it was interesting to observe that both control and Enrofloxacin poults had an increase in Gammaproteobacteria, but Clostridia and Bacilli were decreased in Enrofloxacin birds when compared with control or poults treated with the probiotic (Table 4).

**Table 4:** **Phylum distribution (cumulative% lowest common ancestor) and class direct assignment in % for all ceca samples of turkey poults following prophylactic administration of FloraMax-B11^®^ or Enrofloxacin**.

	Control + SH	FloraMax-B11^®^ + SH	Enrofloxacin 50 mg/kg + SH
**Phylum**
Firmicutes	42 ± 10^b^	55 ± 8^a^	9 ± 4^c^
Bacteroidetes	19 ± 6^a^	23 ± 4^a^	10 ± 2^b^
Proteobacteria	29 ± 4^a^	18 ± 5^b^	31 ± 3^a^
**Class**
Gammaproteobacteria	15.07 ± 2.58^a^	6.16 ± 0.083^b^	24.95 ± 2.76^a^
Clostridia	5.01 ± 2.22^a^	4.25 ± 1.30^a^	2.40 ± 0.04^b^
Bacilli	3.05 ± 0.01^a^	4.21 ± 0.01^a^	1.11 ± 0.06^b^

*Experiment 2*.

*^a,b^Superscripts within rows indicate significant difference at P < 0.05, n = 6*.

## Discussion

Considerable scientific evidence has shown that the use of certain antibiotics increases enteric colonization of antibiotic-resistant strains of enteric pathogens in domestic animals (28–32). Because some of these pathogens are extremely resistant to many antibiotics and are capable of rapidly developing resistance when exposed (7, 13, 14), antibiotic prophylaxis or treatment has been reported to actually increase the occurrence and severity of these infections in commercial poultry (33, 34). In addition, the lack of effect of these antibiotics in resistant enteropathogens, some researchers have shown that antibiotics can actually cause disruption in the microbiome (35), accompanied with reduction of short chain fatty acids (36, 37) and increased luminal pH in the distal gastrointestinal tract (38). In the present study, we evaluate the management practice in certain countries of using five times the recommended dose of Enrofloxacin in neonatal chickens and turkey poults for five consecutive days after placement, and look at their susceptibility to salmonellae infections 24 h after treatment. In trial 1 of Experiment 1, chickens treated with either 25 or 50 mg/kg of Enrofloxacin were more susceptible to SE organ invasion when compared with control non-treated chickens. In addition, chickens treated with 50 mg/kg of Enrofloxacin in trial 1 and both Enrofloxacin doses in trial 2 had a significant increase in total SE cfu in cecae when compared with control chickens, suggesting that this management practice performed in poor antimicrobial stewardship countries, increased susceptibility to SE infections in broiler chickens.

*Salmonella* Heidelberg is among the top three *Salmonella* serovars isolated from humans when poultry products were linked to the infection (39–42). Furthermore, SH resistant to various antimicrobial agents has been isolated from domestic animals (43–45). In Experiment 2, our results are in agreement with previous publications from our laboratory, showing not only the low invasiveness of SH for internal organs, but also effectiveness of FloraMax-B11^®^ in reducing SH intestinal colonization in turkey poults (46). Published studies have also shown that FloraMax^®^-B11 increased colonization resistance to *Salmonella* spp. infections (47–51), reduces idiopathic diarrhea in commercial turkey brooding houses (52), as well as increased performance and reduced costs in poultry production (53, 54). In the present study, it was remarkable to observe that poults treated with 50 mg/kg of Enrofloxacin were more susceptible to SH colonization and that this effect was associated with a significant reduction in the total number of LAB. Poults treated with 50 mg/kg of Enrofloxacin showed a significant increase in BW and BWG, however, this group also showed a significant increase in gut permeability. Metagenomic analysis of cecal content using the MEGAN software can be used to interactively analyze and compare metagenomic and metatranscriptomic data, thereby providing a percent identity filter that can be used to enforce the following levels of percentage sequence identities for an assignment at a given taxonomic level (25). In Experiment 2, poults treated with Enrofloxacin had a lower proportion of Firmicutes and Bacteroidetes, suggesting that the broad spectrum of Enrofloxacin had a profound impact upon the microbiome. Interestingly, these poults had the highest proportion of Proteobacteria (similar to control). Such a high dose of antibiotic also had a significant increase in Gammaproteobacteria. Changes in the proportion of phylum and class were associated with higher SH intestinal colonization since *Salmonella* belongs to phylum Proteobacteria, class Gammaproteobacteria. Furthermore, poults treated with Enrofloxacin had lower proportions of Clostridia and Bacilli when compared with control or probiotic poults. Antibiotics administered in low doses have been widely used as growth promoters in poultry for over half a century. However, the exact mechanisms for this effect are elusive. Similarly, there are no reports that have described the impact of Enrofloxacin at low or high therapeutic dose on the microbiome or metabolomics in poultry. This is the first report that describes profound changes in microbiome of turkey poults that received a high dose of Enrofloxacin, shifting it and making them more susceptible to a SH experimental challenge.

By contrast, poults that received the probiotic had the highest proportion of Firmicutes and Bacteroidetes, but the lowest amount of Proteobacteria. These birds also showed significant reduction in Gammaproteobacteria, but similar to the control group, a higher proportion in Clostridia and Bacilli. The shift in these bacterial populations had a positive effect on reducing SH colonization following challenge and confirms our previous research (46).

The results of these experiments suggest that, five times the recommended dose of Enrofloxacin, a broad-spectrum antibiotic can have a negative effect on the microbiome that may be responsible for an enhancement of SH colonization, which has been previously demonstrated with other enteropathogens (4, 28, 29, 31, 32). The mechanism of antibiotic-altered resistance was not investigated in the present study. However, regardless of the mechanism involved, increased susceptibility of turkey poults to *Salmonella* was observed in two experiments following Enrofloxacin treatment. Furthermore, based on the microbiota changes following fluoroquinolone administration, including the increase in Proteobacteria, these results suggest that this practice may predispose to other infectious diseases that will further require the use of additional antibiotics and broaden the selection of antimicrobial resistance. Acquisition of resistance to fluoroquinolones has been reported to be a multifaceted process, which includes spontaneous point mutations that result in amino acid substitutions within the topoisomerase subunits GyrA, GyrB, ParC, or ParE, reduced expression of outer membrane porins, overexpression of multidrug efflux pumps, and/or plasmid-mediated quinolone resistance (1, 2, 7, 13, 15, 55, 56). It is remarkable to contemplate that the alarming incidence of certain enteric pathogens is associated with the indiscriminate use of some antibiotics in animal agriculture in some countries (42, 45, 57–61). Since poultry products have been identified as important reservoirs of human infections, this is a growing public health concern. Given that fluoroquinolones and other antibiotics are over used in animal production, any effort to diminish the risk of resistance is crucial. The results of the present study and of previous investigations involving antibiotics and other enteropathogens suggest that prophylactic utilization of some antibiotics in poultry increase the susceptibility to salmonellae colonization and organ invasion. Therefore, antibiotics should be limited to infections of specific bacteria with known antibiotic sensitivity. In addition, our findings also confirm previous studies suggesting that the use of alternatives, such as probiotics, can be an effective tool in controlling salmonellae infections.

## Author Contributions

EM-B, NC, JL-T, and VL: conception and design, acquisition of data, and drafting of manuscript. XH-V, JL, BH, GT: drafting the article and revising it critically for important intellectual content. OP-R, RM-G, VP-G, AW, MB, BM, KT, and LG: acquisition of data. BH and GT: analysis and interpretation of data, drafting of manuscript, and approval of the version to be submitted and any revised version.

## Conflict of Interest Statement

The authors declare that the research was conducted in the absence of any commercial or financial relationships that could be construed as a potential conflict of interest.
